# mRNA expression profiles in muscle-derived extracellular vesicles of Large White and wild boar piglets reveal their potential roles in immunity and muscle phenotype

**DOI:** 10.3389/fvets.2024.1452704

**Published:** 2024-10-03

**Authors:** Naixiang Yu, Xiaolong Chang, Jianchao Hu, Jianjun Li, Junwu Ma, Lusheng Huang

**Affiliations:** National Key Laboratory of Pig Genetic Improvement and Germplasm Innovation, Jiangxi Agricultural University, Nanchang, Jiangxi, China

**Keywords:** exosomes, extracellular vesicles, mRNA, muscle, pig

## Abstract

**Introduction:**

Extracellular vesicles (EVs) known for their pivotal role in intercellular communication through RNA delivery, hold paramount implications for understanding muscle phenotypic variations in diverse pig breeds.

**Methods:**

In this study, we compared the mRNA expression profiles of *longissimus dorsi* muscles and muscle-derived extracellular vesicles (M-EVs), and also examined the diversity of enriched genes in M-EVs between weaned wild boars and commercial Large White pigs with respect to their numbers and biological functions.

**Results:**

The results of the study showed that the variation in the expression profiles of mRNAs between muscles and M-EVs was much greater than the variability between the respective breeds. Meanwhile, the enrichment trend of low-expressed genes (ranked <1,000) was significantly (*p*-value ≤ 0.05) powerful in M-EVs compared to highly expressed genes in muscles. In addition, M-EVs carried a smaller proportion of coding sequences and a larger proportion of untranslated region sequences compared to muscles. There were 2,110 genes enriched in M-EVs (MEGs) in Large White pigs and 2,322 MEGs in wild boars, with 1,490 MEGs shared interbreeds including *cyclin D2* (*CCND2*), which inhibits myogenic differentiation. Of the 89 KEGG pathways that were significantly enriched (*p*-value ≤ 0.05) for these MEGs, 13 unique to Large White pigs were mainly related to immunity, 27 unique to wild boars were functionally diverse but included cell fate regulation such as the Notch signaling pathway and the TGF-beta signaling pathway, and 49 were common to both breeds were also functionally complex but partially related to innate immunity, such as the Complement and coagulation cascades and the Fc gamma R-mediated phagocytosis.

**Discussion:**

These findings suggest that mRNAs in M-EVs have the potential to serve as indicators of muscle phenotype differences between the two pig breeds, highlighting the need for further exploration into the role of EV-RNAs in pig phenotype formation.

## 1 Introduction

Exosomes are small extracellular vesicles (EVs) with a diameter of 50–150 nm, originating from endosomes and released by cells (1). They have the ability to mediate intercellular communication and influence many aspects of cell biology such as growth, development, immunity, and metabolism (2). The intricate biological functions of EVs hinge on their capability to transfer cargoes, including RNA, and these cargoes in EVs hold immense potential as biomarkers for physiological state of the body because their composition and abundance depend on the cell state (3). Importantly, the content in EVs and their RNA cargoes may be influenced by microenvironment and the inherent biology of the cells (2).

Among the RNA cargoes in EVs, messenger RNA (mRNA) has garnered particular attention due to its potential for direct translation in recipient cells. The directed evidence of this possibility came from the fluorescent labeling of EVs mRNA in the co-culture between glioblastoma and HEK293 T cells (4). Moreover, it has also been reported that EV-associated mRNA may regulate the stability, localization and translation of endogenous mRNA in target cells (5).

Skeletal muscle is the main organ for glucose uptake and storage, which contributes to glucose and energy homeostasis (6), and also can secret EVs that function in an autocrine, paracrine, and endocrine manner (7). On the one hand, muscle-derived EVs can mediate communication between muscle cells. For example, EVs released by myotubes can inhibit the proliferation of myoblasts and induce the expression of the early differentiation maker gene (8). Myogenic progenitor cells produced after activation of satellite cells release EVs carrying miR-206, which inhibits the deposition of extracellular matrix collagen by interstitial fibrogenic cells, promoting muscle fiber hypertrophy (9). EVs release from myotube under oxidative stress simulated by H_2_O_2_ can reduce myotube diameter and promote myoblast proliferation (10). On the other hand, muscle-derived EVs also mediate communication between muscle cells and others. For example, EVs derived from skeletal muscle of mice fed with 20% palmitate induced proliferation of islet MIN6B1 cells (11). Skeletal muscle under mechanical overload releases EVs carrying miR-1, which can promote lipolysis after absorption by epidydimal white adipose tissue (12).

Pigs, a pivotal meat source for humans, trace their lineage back to domesticated wild boars approximately 9,000 years ago (13). Substantial genetic modification resulting from prolonged domestication and selective breeding distinguish commercial pig breeds from their wild boar ancestors, engendering various phenotypic differences (14–16). For instance, wild boars possess more slow-twitch oxidative (I) and fast-twitch oxidative glycolytic (IIA) muscle fibers, and less fast-twitch glycolytic (IIB) fibers, leading to darker, less tender and leaner meat compared to commercial pig breeds (17). The genetic and muscle phenotypic variation between wild boars and domestic pigs suggest the existence of biological differences in their muscle cells, which may be manifested in the RNA contents of muscle-derived extracellular vesicles. Yet, the extent to which the RNA expression profile in muscle-derived extracellular vesicles co-evolves with muscle transcriptome and its implications for formation of breed-specific muscle phenotypes remains unclear.

Therefore, the aim of this study was to compare the differences of mRNA expression profile characteristics between porcine muscles and porcine muscle-derived extracellular vesicles, and to explore the discrepancy of genes enriched in the muscle-derived extracellular vesicles of wild boars and Large White pigs, as well as their potential physiological functions. In accordance with the recommendations of the International Society for Extracellular Vesicles (ISEV), we use the term “EVs” to refer to exosomes in this paper (18).

## 2 Materials and methods

### 2.1 Sample collection

*Longissimus dorsi* (LD) muscle samples were collected from four healthy and post-weaned piglets including Large White (one male and one female) from a farm of Zheng Bang Group (Jiangxi, China) and wild boar (one male and one female) from forests in southern China (Supplementary Table S1). All samples were temporarily stored at liquid nitrogen and finally stored in a refrigerator at −80°C.

### 2.2 EVs isolation

EVs were isolated from *longissimus dorsi* samples using the protocol established previously by Vella et al. (19), with minor modification. A small (~200 mg) piece of tissue was briefly sliced on dry ice and then incubated in the dissociation mixture (contained enzymes H, R, A, and Buffer A) which was prepared based on the Human Tumor Dissociation Kit (130-095-929, Miltenyi Biotec, Germany) for 10–15 min at 37°C. The dissociated tissue filtered through a 70 μm filter gently for twice to remove residual tissues. Then suspension was centrifuged (300 g for 10 min) at 4°C, and the supernatant was collected and subsequently centrifuged (2,000 g for 10 min) at 4°C. Cell-free supernatant was collected and centrifuged (10,000 g for 20 min) at 4°C, then filtered through a 0.22 μm filter for further depletion of cell debris. The collected suspension was then processed by ultracentrifugation (UC) at 150,000 g for 2 h at 4°C. The pellet was resuspended in 1 mL 1x phosphate-buffered saline (PBS) and further purified using Exosupur® columns (Echobiotech, China). Fractions were concentrated to 200 μL by 100 kDa molecular weight cut-off Amicon® Ultra spin filters (Merck, Germany).

### 2.3 Nanoparticle tracking analysis (NTA)

EVs with concentrations between 1×10^7^/ml and 1×10^9^/ml were examined using the ZetaView PMX 110 (Particle Metrix, Meerbusch, Germany) equipped with a 405 nm laser to determine the size and quantity of particles isolated.

### 2.4 Transmission electron microscope (TEM)

The 10 μL EVs solution was placed on a copper mesh and incubated at room temperature for 1 min. After washing with sterile distilled water, the EVs were contrasted by uranyl acetate solution for 1 min. The sample was then dried for 2 min under incandescent light. The copper mesh was observed and photographed under a transmission electron microscope (H-7650, Hitachi Ltd., Tokyo, Japan).

### 2.5 Western blot analysis

Utilize the RIPA Lysis Buffer (CW2333, Cwbio IT Group, China) to efficiently lyse muscle tissue and extract proteins. Subsequently, employ the Micro BCA assay kit (23,235, Thermofisher, USA) to quantify the protein concentration and EVs directly leverage the BCA assay kit to assess their own protein concentrations. Based on the BCA results, accurately calculate the protein loading quantity for muscle tissue and EVs, aiming for 50 μg per line. Subsequently, proportionately add 5 x Sodium dodecyl sulfonate (SDS) Loading Buffer, thoroughly mix via vortex shaking, and denature the protein in 95°C water bath for 5 min. Once denatured, perform electrophoresis in a 10% SDS-PAGE gel. To efficiently transfer the target protein to a 0.2 μm Polyvinyli-dene Fluoride (PVDF) membrane, utilize a constant current of 200 mA for 1 h. Next, immerse the PVDF membrane, containing the target protein, in a 5% skim milk pow-der solution and allow it to block at room temperature for 1–2 h. Subsequently, incubate the membrane with the following rabbit polyclonal antibodies: HSP70 (10995-1-AP, Proteintech, USA), Alix (D262028, Sangon Biotech, China), TSG101 (381,538, ZenBio, China), and Calnexin (10427-2-AP, Proteintech, USA). Begin the incubation at room temperature for 10 min, then continue overnight at 4°C. After the overnight incubation, add the secondary antibody, HRP-conjugated Affinity Goat An-ti-Rabbit IgG (H + L) (SA00001-2, Proteintech, USA), and incubate on a shaker at room temperature for 1 h. Finally, utilize a chemiluminescence gel imaging system (Tanon-5200Multi, Tanon, China) to capture the imaging of the protein bands.

### 2.6 Total RNA extraction from EVs

Total RNA extraction from EVs using miRNeasy Serum/Plasma Kit (217,184, QI-AGEN, Germany) according to the manufacturer’s instruction with minor modification. 700 μL QIAzol Lysis Reagent was added to the EVs, mixed by vortexing and then incubated at room temperature for 5 min. 140 μL chloroform/isoamylalcohol (24:1) was added, shaken vigorously for 15 s, and continued to incubate at room temperature for 2–3 min. Centrifuge for 15 min at 12,000 xg at 4°C and the upper aqueous phase was collected. 1.5 volumes of 100% ethanol was added and mixed. Pi-pet up to RNeasy MinElute spin column for purification, and then the spin column was washed by adding 700 μL Buffer RWT and 500 μL Buffer RPE. Centrifuge for 2 min at 12,000 xg to dry membrane of the spin column. The spin column was placed into a new collection tube and 20 μL RNase-free water was added to the center of the spin column membrane, and centrifuge (12,000 xg, 2 min) to elute the RNA. Finally, RNA quantity and quality were assessed using Agilent 2,100 bioanalyzer (Thermo Fisher Scientific, MA, USA).

### 2.7 Total RNA extraction from muscles

Total RNA extraction from muscles using TRIzol Reagent (15,596,026, Invitrogen, USA) following the manufacturer’s instruction. Take appropriate amount of tissue samples, grind the tissue samples into powder with liquid nitrogen and transfer into the 1.5 mL TRIzol lysis buffer. Stand flat for 5 min for the fully lysis of cells. Centrifuge the tissue samples after grinding and crushing at 12000 xg for 5 min in 4°C. Transfer the supernatant to the centrifuge tube with 300 μL Chloroform/isoamyl alco-hol (24:1) mix thoroughly by upside down violent shaking. Transfer the supernatant to the 1.5 mL centrifuge tube, add 2/3 volume of Isopropyl alcohol. Gently invert and mix, place in −20°C refrigerator for more than 2 h. Centrifuge at 17500 xg for 25 min in 4°C. Discard the supernatant, wash with 0.9 mL 75% ethanol, suspend the precipita-tion by inverting. Centrifuge at 17500 xg for 3 min in 4°C, Discard the supernatant, centrifuge for a short time and use the tip to remove the residual liquid, air dry for 3–5 min. Dissolve the precipitation with 20 ~ 200 μL RNase-free water. Finally the RNA integrity was assessed via Agilent 2,100 bioanalyzer with RIN ≥ 7.

### 2.8 cDNA library construction and sequencing of EVs

The cDNA library of EVs were constructed using BGI Optimal Dual-mode mRNA Library Prep Kit (LR00R96, BGI, China) following the manufacturer’s protocol. RNase H was used to remove the rRNA, DNase I was used to digest DNA in total RNA. Purified RNA from previous steps was fragmented into small pieces with fragment buffer at appropriate temperature. Then, First-strand and second-strand cDNA were generated in Master Mix by PCR. The double-stranded cDNA ends were repaired, and the 3 ‘end was added with a single ‘A’ base. Subsequent, adaptor was connected to the cDNA. The products were amplified, denatured and cyclized using PCR to create the single strand circle cDNA library. Finally, constructed libraries were sequenced on the MGISEQ-2000 System (BGI-Shenzhen, China) to generate paired-end reads.

### 2.9 cDNA library construction and sequencing of muscles

The BGI Optimal Dual-mode mRNA Library Prep Kit (LR00R96, BGI, China) was employed to construct a muscle cDNA library, adhering strictly to the manufacturer’s protocol. PolyA-mRNAs were enriched using oligo(dT)-attached magnetic beads, fragmented, and subsequently utilized for the synthesis of first- and second-strand cDNA. These double-stranded cDNA fragments underwent end-repair, followed by the addition of a single ‘A’ nucleotide to the 3′ ends of the blunt fragments. Post-adaptor ligation, the cDNAs were amplified via PCR, resulting in single-stranded PCR products through denaturation. Additionally, single-stranded cyclized products were generated through PCR program for circularization, while uncyclized linear DNA molecules were digested. Ultimately, the constructed libraries were sequenced on the MGISEQ-2000 System (BGI-Shenzhen, China) to yield paired-end reads.

### 2.10 Analysis of total expression profiles of M-EVs and muscle tissue

Errors, adapters and low-quality reads in the row data were filtered using SOAPnuke v1.5.2 (20), and clean data was generated. The clean data was aligned to pig reference genome (Sscrofa11.1) using hisat2 v2.2.1 (21), and reads count was calculated by StringTie v2.1.4 (22), and then reads count was standardized to FPKM (fragments per kilobase million). Genes with FPKM values less than 1 in both muscles and M-EVs were eliminated (23). Differentially expressed analysis was carried out via DESEq2 between M-EVs and muscles (24), and then genes in the M-EVs with *P*adj (adjusted *P*) ≤ 0.05 and greater than or equal to 2 fold of muscle tissue were considered to MEGs (genes enriched in M-EVs). KEGG pathway enrichment analysis of MEGs were performed using KOBAS-I (25).

The distribution of reads in different gene regions of M-EVs and muscles were evaluated by Read_distribution.py from RSeQC v5.0.1 (26). Meanwhile, featureCounts were utilized to quantify gene expression levels based on the features including CDS region, 5’UTR, and 3’UTR regions, and genes with FPKM values equal to 0 in both muscles and M-EVs were eliminated (27). mRNA with full length potential was defined as reads were all detected in the CDS, 5’UTR, and 3’UTR regions.

Principal component analysis was performed using prcomp function of R, volcano plots were drew using ggpubr (28) package of R and remaining analysis results were visualized ggplot2 (29) package in R. Furthermore, the plot of KEGG network were drew using Cytoscape (30). The visualization of genomic alignment reads using Integrative Genomics Viewer (IGV) v2.8.7 (31).

### 2.11 Validation of RNA sequencing by quantitative real-time polymerase chain reaction

For validation, 13 DEGs were randomly selected and primers were synthesized by Sangon Biotech (Supplementary Table S2). The rest total RNA of muscles and M-EVs after sequencing were used as template for reverse-transcribed into cDNA using the PrimeScript™ RT reagent Kit with gDNA Eraser (Takara, Japan), and then the quantitative PCR was executed with GAPDH as reference gene using the TB Green® Premix Ex Taq™ II (Takara, Japan) on the 7900HT Fast Real-Time PCR System (Applied Biosystems, USA). Finally, the relative gene expression levels were determined using the 2^−ΔΔCT^ method.

### 2.12 Statistical analysis

Numbers of experimental replicates are given in the figure legends. This study utilized the R language for statistical analysis. The Pearson correlation analysis was employed to examine the correlation between data from two groups. Analysis of Variance (ANOVA) and Least Significant Difference (LSD) multiple comparison were used to analyze the differences among multiple (greater than 2) groups. A *p*-value or adjusted *p*-value (false discovery rate) less than 0.05 was considered statistically significant.

## 3 Results

### 3.1 Characterization of EVs isolated from muscles

**Figure 1:**
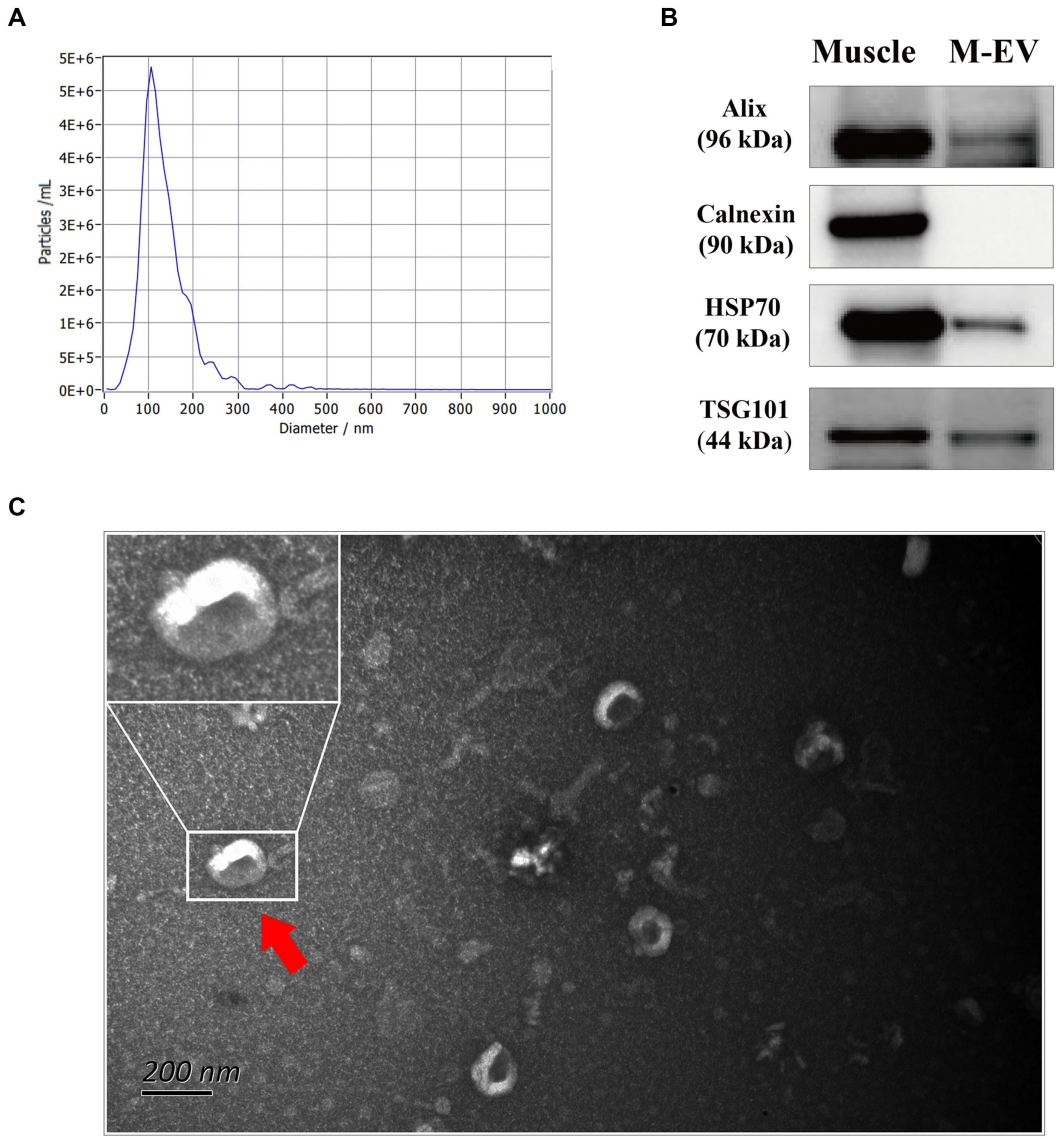
Characterization of M-EV from one pig via nanoparticle trafficking analysis (NTA), western blotting (WB) and transmission electron microscopy (TEM). **(A)** Size distribution determination of M-EV using NTA. **(B)** Expression of M-EV protein makers including TSG101, Alix, HSP70 and Calnexin. **(C)** Image of M-EV in TEM. The scale bars represent 200 nm and red arrows indicate EV. M-EV, muscle-derived EV.

In the present study, muscle-derived EV (M-EV) isolated from *longissimus dorsi* (LD) of one pig was executed characterization through nanoparticle trafficking analysis (NTA), Western blotting and transmission electron microscopy (TEM) according to the ISEV guidelines for studies of extracellular vesicles (18). NTA confirmed a peak particle size of M-EV close to 100 nm (Figure 1A). Western blotting analysis further detected the presence of extracellular vesicles markers (TSG101, Alix, HSP70) in M-EV, while the negative extracellular vesicles marker (Calnexin) was not detected (Figure 1B; Supplementary Figure S1). Additionally, the results of TEM showed that M-EV displayed a classic cup shape (Figure 1C).

### 3.2 Stronger tendency for low expressed genes in muscle tissue to be enriched in muscle-derived EVs

**Figure 2:**
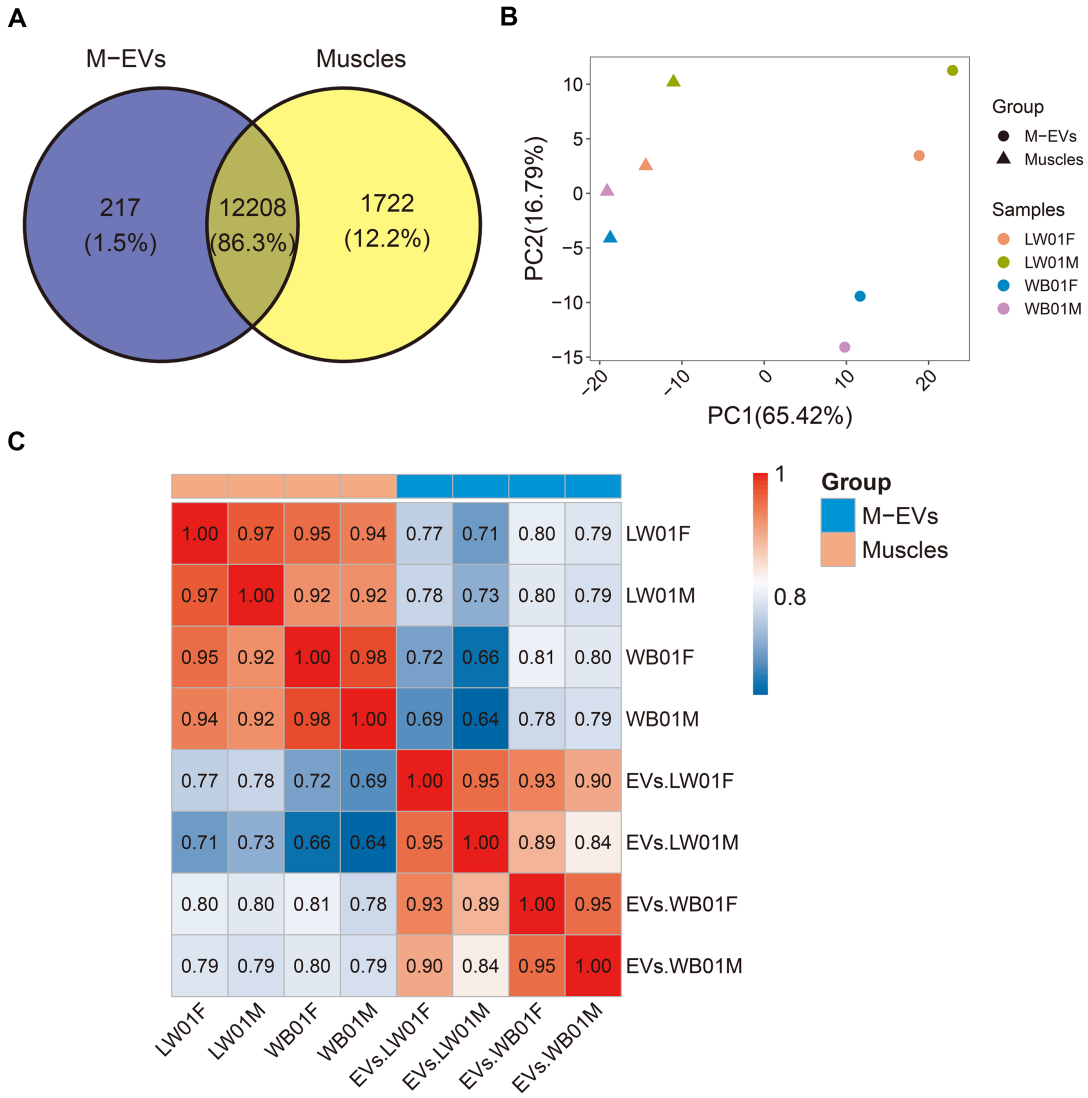
Divergence of gene expression profiles between M-EVs and muscles. **(A)** Venn plot of overlapped genes between muscles and M-EVs (*n* = 4). **(B)** Principal component analysis of gene FPKM values after log10 transformation in M-EVs and muscles (*n* = 4). **(C)** Pearson correlation heatmap of gene FPKM values after log10 transformation between M-EVs and muscles (*n* = 4). M-EVs, Muscle-derived EVs.

To explore the mRNA profiles of muscles and M-EVs, RNA sequencing (RNA-seq) were carry out. As shown in Table 1, the RNA-seq data yield an average of ~52,173,369 clean reads for M-EVs and ~ 40,075,017 clean reads for muscles. However, the genome mapping rates of reads for M-EVs ranged from 61.2 to 72.6%, which were lower than the rates observed in muscles (ranged from 90.2 to 92.6%). Among all the detected genes with FPKM greater than 0, 13,930 were both identified in the muscle samples and 12,425 were both identified in the M-EVs samples (Table 1). Meanwhile, 12,208 genes were overlapped in the 13,930 gens of the muscles and the 12,425 genes of the M-EVs (Figure 2A). For further analysis, gene with FPKM values less than 1 in both muscles and M-EVs was excluded. Subsequently, a principal component analysis (PCA) of gene expression profiles was conducted, which revealed a greater variance between M-EVs and muscles compared to the divergence among breeds, as well as indicating the possibility that RNA profiles in M-EVs co-evolve with the muscle transcriptome (Figure 2B). The result of PCA was further confirmed by the heatmap of expression correlation among samples (Figure 2C).

**Table 1:** Basic information of clean data in muscles and M-EVs.

Class	Breeds	Samples	Clean reads	Q30 (%)	Mapping ratio (%)	Genes (FPKM > 0)
Muscles	Large White pigs	LW01F	40,096,681	91.0	91.8	15,206
		LW01M	40,007,771	90.9	92.6	15,607
	wild boars	WB01F	40,111,944	90.1	90.2	14,856
		WB01M	40,083,675	91.0	91.5	15,179
M-EVs	Large White pigs	LW01F	52,692,130	90.6	72.6	14,206
		LW01M	49,855,313	87.5	61.2	14,005
	wild boars	WB01F	49,230,509	90.3	67.0	13,637
		WB01M	56,915,525	90.7	71.3	13,681

**Figure 3:**
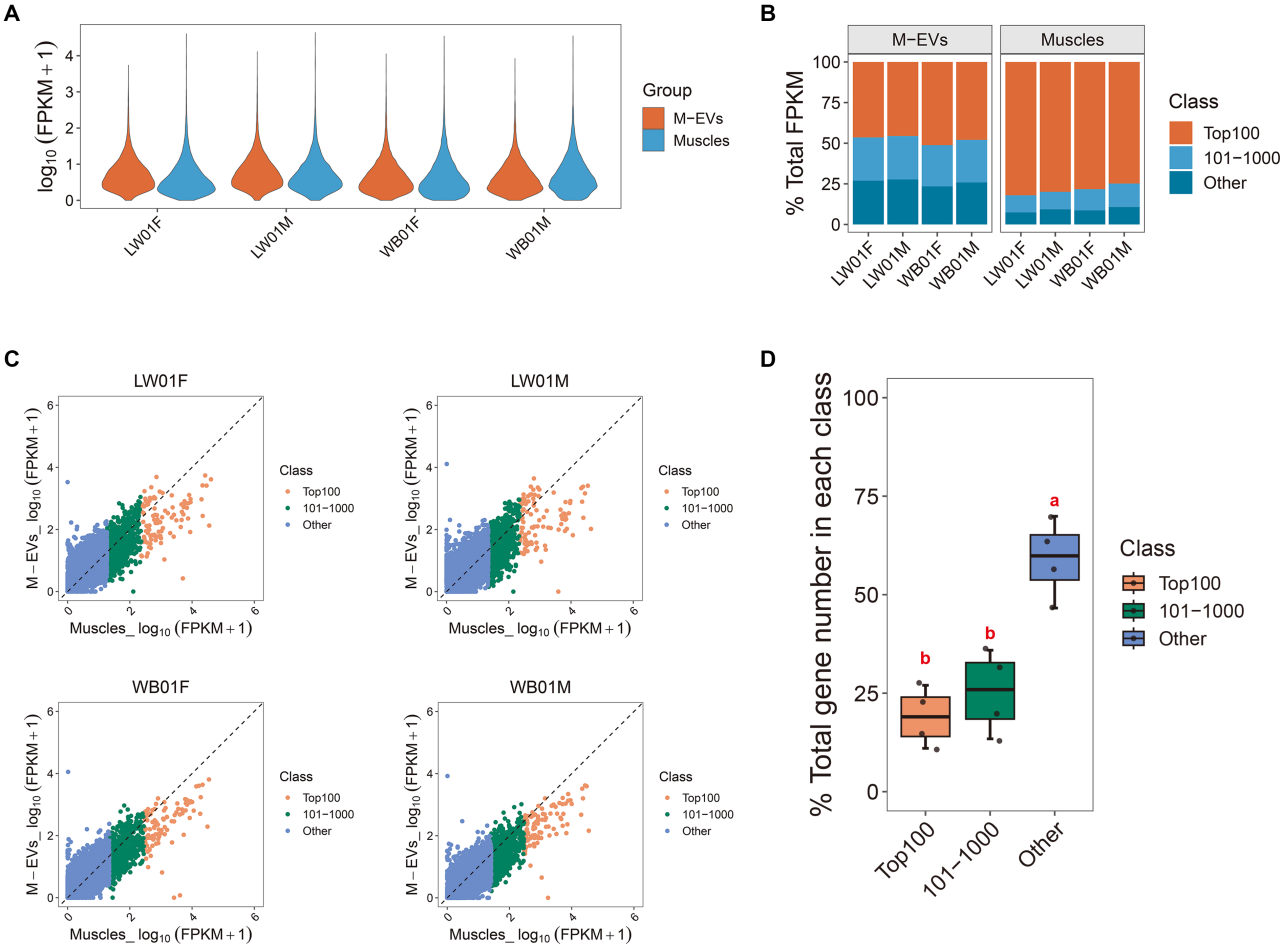
Reasons for divergence in gene expression profiles between M-EVs and muscles. **(A)** Violin plot of gene expression in M-EVs and muscles (*n* = 4). **(B)** Diversity histogram of the composition in the M-EVs and muscles expression profiles (*n* = 4). **(C)** The genes detected in muscles and their corresponding expression in M-EVs (*n* = 4). **(D)** The percentage of genes detected in muscles with maintained or increased expression in M-EVs (*n* = 4). The dashed line represents that the expression of gene detected in the muscles is equal to the corresponding expression in the M-EVs; Different lowercase letters indicate significant differences (*p*-value ≤0.05); The “Class” covers three muscle gene types: Top100 (top 100 abundance), 101–1,000 (101st-1000th abundance), and Other (below 1000th abundance); M-EVs, Muscle-derived EVs.

To elucidate the disparities in expression profiles between muscles and M-EVs, we characterized gene expression using a violin plot. The result showed that the top abundance genes in muscles had higher FPKM values than those in M-EVs (Figure 3A). Furthermore, an average 90.94% of the total FPKM could be attributed to the top 1,000 most abundant genes in muscles, whereas only an average 73.99% of the total FPKM was related to the top 1,000 most abundant genes in M-EVs (Figure 3B). On average, only 20% of the 100 most abundant genes in muscles maintained or increased their expression in M-EVs, and only 25% of the 101–1,000 most abundant genes in muscles maintained or increased their expression in M-EVs (Figures 3C,D). Interestingly, compared with these two types of genes, there was a higher proportion of other genes with low expression in muscles remained unchanged or increased their expression in M-EVs (an average of 60%), and they were significantly different (Figures 3C,D).

These results indicate a strong tendency for genes that are lowly expressed in muscles to be enriched in M-EVs, suggesting that these genes may function through M-EVs and that there is a mechanism that promotes active enrichment of mRNA in M-EVs.

### 3.3 Muscle-derived EVs have a preference for carrying UTR sequences of mRNAs

**Figure 4:**
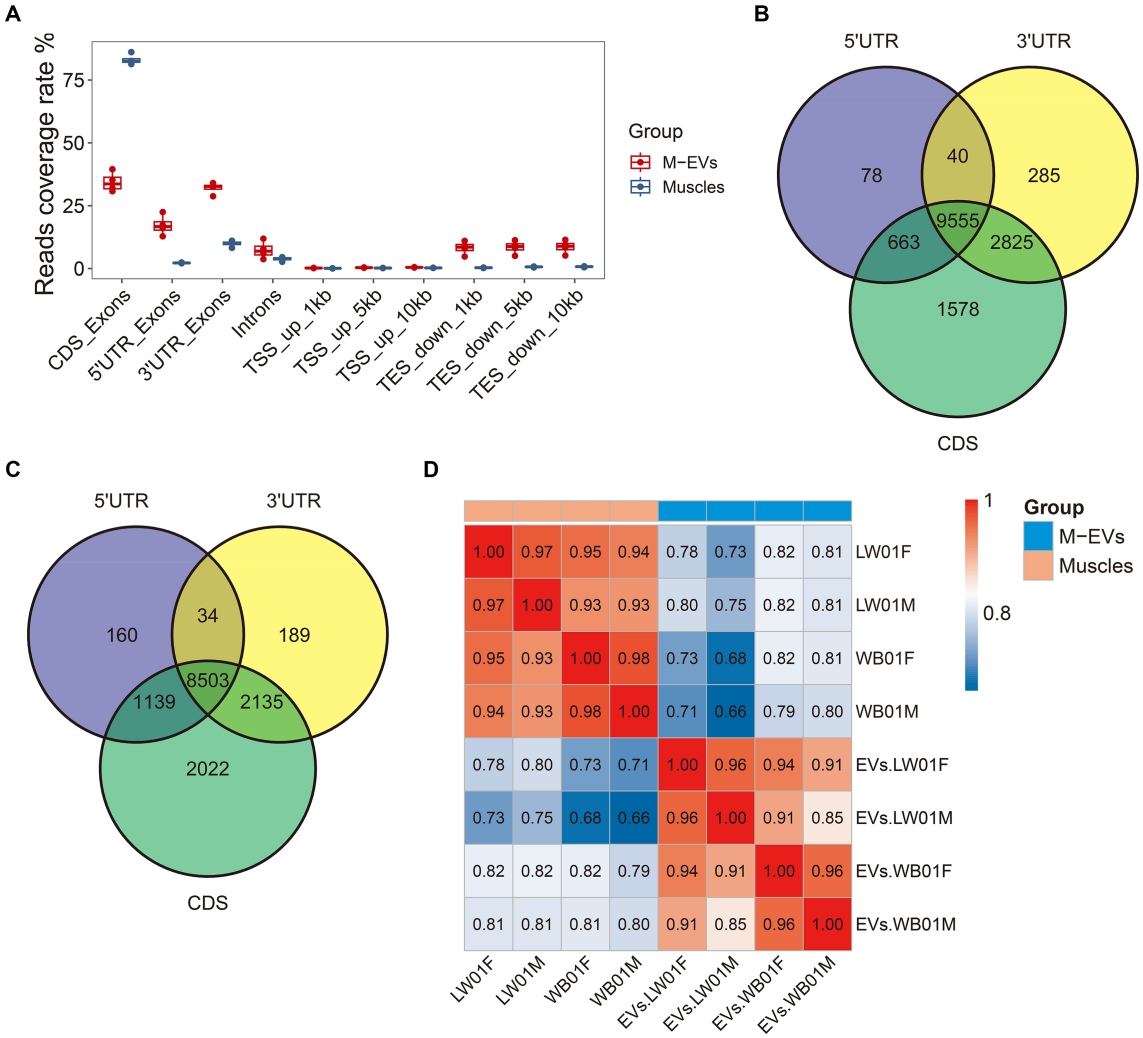
Assessment of mRNA integrity in M-EVs and muscles. **(A)** Percentage coverage of reads in different regions of the gene (*n* = 4). The number of overlapping genes (reads count >0) between samples are detected by individual quantification in three different regions from muscles (*n* = 4) **(B)** and M-EVs (*n* = 4) **(C)**. **(D)** Pearson correlation heatmap of FPKM values from gene with full-length potential after log10 transformation between M-EVs and muscles (*n* = 4). The reads coverage rate indicates the ratio between tag count for different regions and total assigned tags; M-EVs, Muscle-derived EVs.

An increasing number of studies have shown that EVs carry mRNAs that are only partially intact, so it is important to distinguish the full-length mRNAs carried by EVs and their expression profiles. The analysis of the reads coverage of different regions on the genome showed that compared with muscle tissue, the reads within coding sequences (CDS) region in M-EVs were significantly reduced, while more reads were aligned to the untranslated region (UTR) region, which confirmed that only part of the mRNA of M-EVs was full-length (Figure 4A). We further used featureCounts to identify mRNAs with full-length potential in muscle tissues and M-EVs (mRNAs with reads all aligned to the region of CDS region, 5’UTR and 3’UTR), and found that 9,555 and 8,503 genes were detected in muscle tissue and M-EVs, respectively (Figures 4B,C). At the same time, 7,477 genes with full-length potential shared between muscle tissue and M-EVs were also detected by StringTie, and the correlation analysis of the expression profiles of these genes detected by StringTie suggested that the full-length potential mRNAs of M-EVs had a significantly different expression pattern from that of muscle tissue (Figure 4D).

### 3.4 *Cyclin D2* is enriched in M-EVs of Large White pigs and wild boars

**Figure 5:**
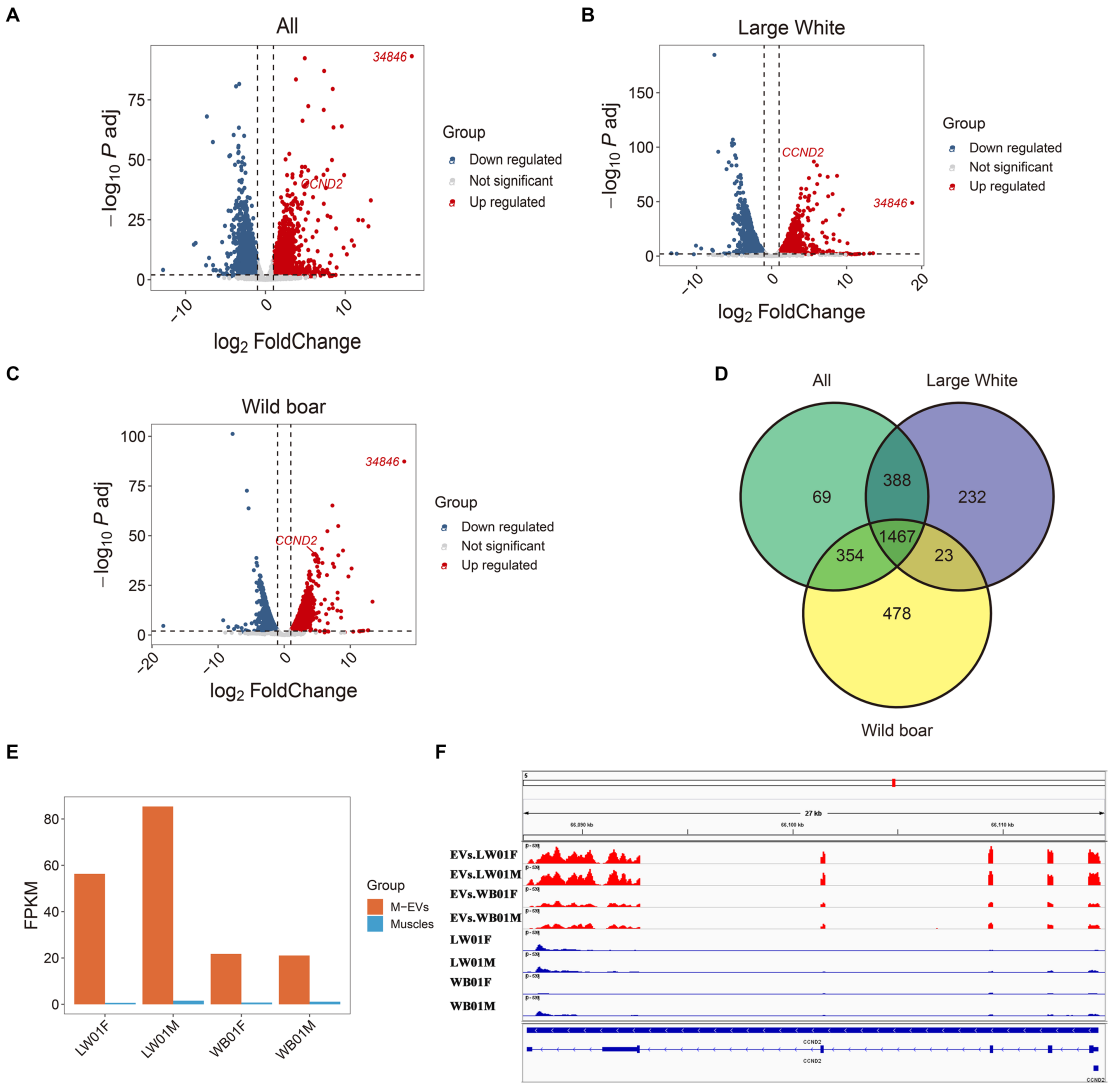
Differential expression between M-EVs and muscles. Volcano plot for differential expression analysis between M-EVs and muscles at three groups: all (*n* = 4) **(A)**, Large White pigs (*n* = 2) **(B)**, and wild boars (*n* = 2) **(C)**. **(D)** Venn plot of number of up-regulated genes in M-EVs at three groups: all (*n* = 4), Large White pigs (*n* = 2) and wild boars (*n* = 2). **(E)** The expression values of *CCND2* (*n* = 4). **(F)** Reads coverage of the *CCND2* (*n* = 4). Up regulated, genes up-regulated in M-EVs; down regulated, genes down-regulated in M-EVs; Not significant, genes with adjusted *p*-value >0.05 or fold change <2; M-EVs, Muscle-derived EVs.

Here, genes enriched in M-EVs (MEGs) were defined as genes with expression levels in M-EVs that were more than 2-fold higher than those in muscle tissue, with an adjusted *p*-value ≤0.05 (Supplementary Tables S3–S5). We then analyzed the different MEGs catalogs at the Large White pigs, wild boars, and overall levels. The results showed that 2,110, 2,322, and 2,278 MEGs were detected in M-EVs from Large White pigs, wild boars, and global level, respectively (Figures 5A–D; Table 2). Moreover, 1,467 genes were shared among the three MEGs sets, and only 69 (3%) genes were present only in the global level containing 2,278 MEGs (Figure 5D). Meanwhile, *Cyclin D2* (*CCND2*) gene with the smallest *p*-value and *34,846* (*ENSSSCG00000034846*) gene with the largest fold change in Large White pig were also detected in the three MEGs sets of Large White pig and global level (Figures 5A–C). Interestingly, *CCND2* was apparently enriched in both wild boars and Large White pigs, with the degree of enrichment varying among breeds (Figure 5E). And the sequence integrity of *CCND2* in M-EVs makes it more fascinating (Figure 5F).

**Table 2:** The number of genes enriched in M-EVs (MEGs) in the Large White pigs, wild boars, and global levels.

Class	Genes used for analysis	MEGs	Ratio
Large White pigs	11,045	2,110	19.1%
Wild boars	10,783	2,322	21.5%
Global levels	9,968	2,278	22.9%

On the one hand, these results confirm that the authenticity of the data remains unchanged during MEGs mining of divided breeds. On the other hand, they preliminarily indicate that the mRNA components of M-EVs exhibit commonalities and specificities among breeds.

### 3.5 Genes enriched in M-EVs of Large White pigs and wild boars are associated with immunity, cell fate, hormones and metabolism

**Figure 6:**
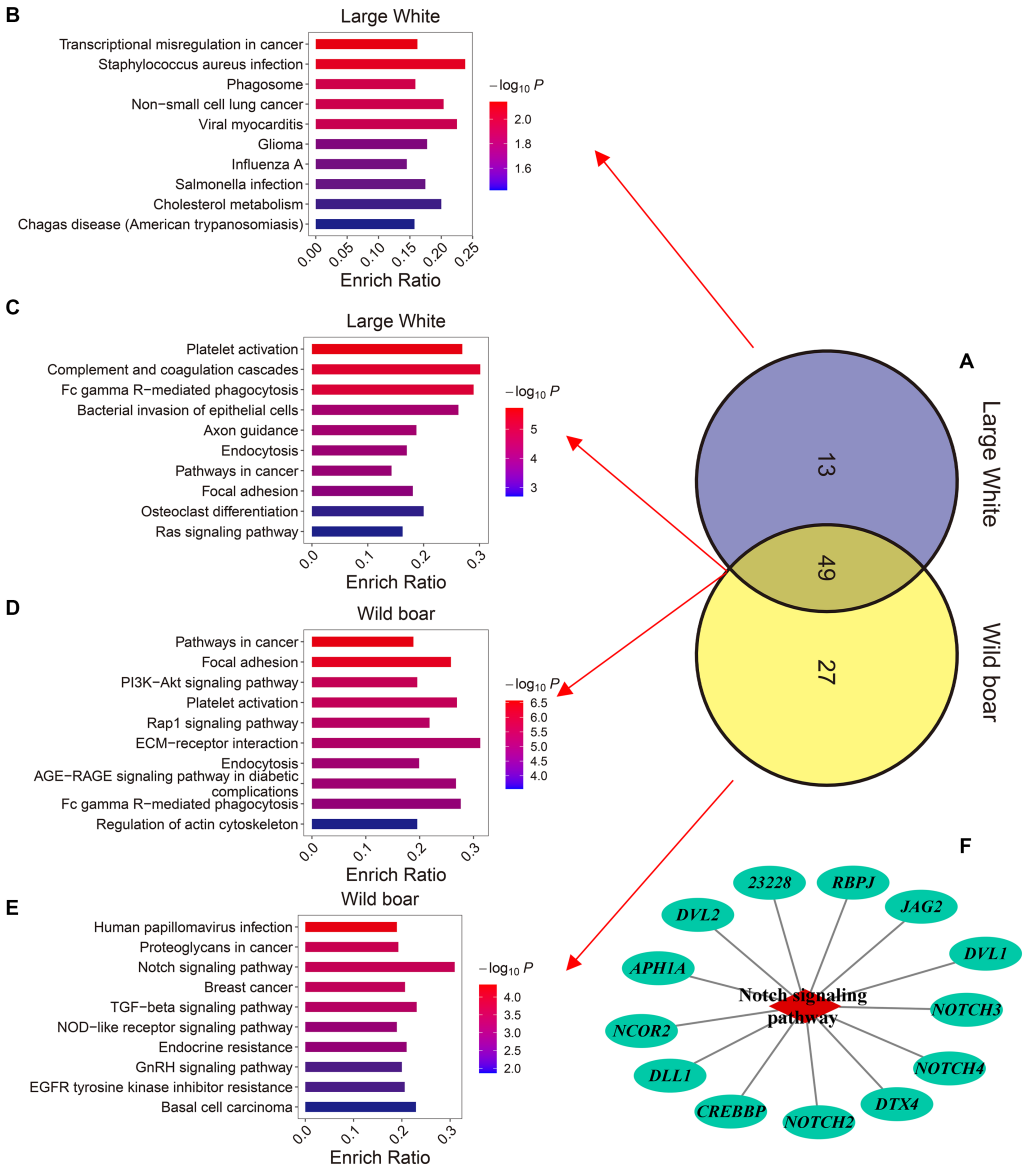
Comparison of the KEGG pathways enriched by the genes up-regulated in M-EVs. **(A)** Venn plot of the number of KEGG enrichment pathways for up-regulated genes in M-EVs from Large White pigs and wild boars. **(B)** The top 10 KEGG pathways uniquely enriched for up-regulated genes in M-EVs from Large White pigs. The top 10 KEGG pathways co-enriched for up-regulated genes in M-EVs from Large White pigs **(C)**, and wild boars **(D)**. **(E)** The top 10 KEGG pathways uniquely enriched for up-regulated genes in M-EVs from wild boars. **(F)** The notch signaling pathway uniquely enriched for up-regulated genes in M-EVs from wild boars. M-EVs, Muscle-derived EVs.

To investigate the biological functions of 2,110 and 2,322 MEGs in Large White pigs and wild pigs, respectively, we conducted Kyoto Encyclopedia of Genes and Genomes (KEGG) enrichment analysis (Supplementary Tables S6, S7). The results showed that the MEGs in Large White pigs were significantly enriched (*p*-value ≤0.05) in 63 KEGG pathways, while the MEGs in wild pigs were significantly enriched (*p*-value ≤0.05) in 76 KEGG pathways. Among these two sets of KEGG pathways, there were 13 pathways specifically enriched by the MEGs in Large White pigs, 27 pathways specifically enriched by the MEGs in wild pigs, and 49 pathways commonly enriched by the MEGs in both species (Figure 6A). The 13 specifically enriched KEGG pathways in Large White pigs are primarily associated with immune regulation, including Transcriptional misregulation in cancer, *Staphylococcus aureus* infection, Phagosome, Non-small cell lung cancer, Viral myocarditis, and other pathways (Figure 6B). The 27 KEGG pathways specifically enriched by the MEGs in wild pig are primarily associated with immune regulation, cell fate (proliferation, differentiation, and apoptosis), hormone regulation, and metabolic regulation. These pathways include Human papillomavirus infection, Proteoglycans in cancer, Notch signaling pathway, Breast cancer, and TGF-beta signaling pathway (Figure 6E). Interestingly, among the specifically enriched pathways by the MEGs in wild pig, the Notch signaling pathway exhibits the highest gene enrichment rate, which is calculated as the ratio of enriched genes to background genes (Figure 6F). It is worth noting that among the 49 KEGG pathways commonly enriched by the MEGs in wild pigs and Large White pigs, 5 pathways are related to innate immunity including Complement and coagulation cascades, Fc gamma R-mediated phagocytosis, Chemokine signaling pathway, B cell receptor signaling pathway, and Natural killer cell-mediated cytotoxicity (Figures 6C,D).

The KEGG analysis results indicate that the potential functions of enriched mRNAs in M-EVs are complex and diverse, with a notable emphasis on immune regulation. And these results also suggest that M-EVs carrying mRNA can reflect both commonalities and heterogeneity in biological functions across different breeds.

### 3.6 The verification of the concordance between quantitative real-time polymerase chain reaction and RNA-Seq findings

**Figure 7:**
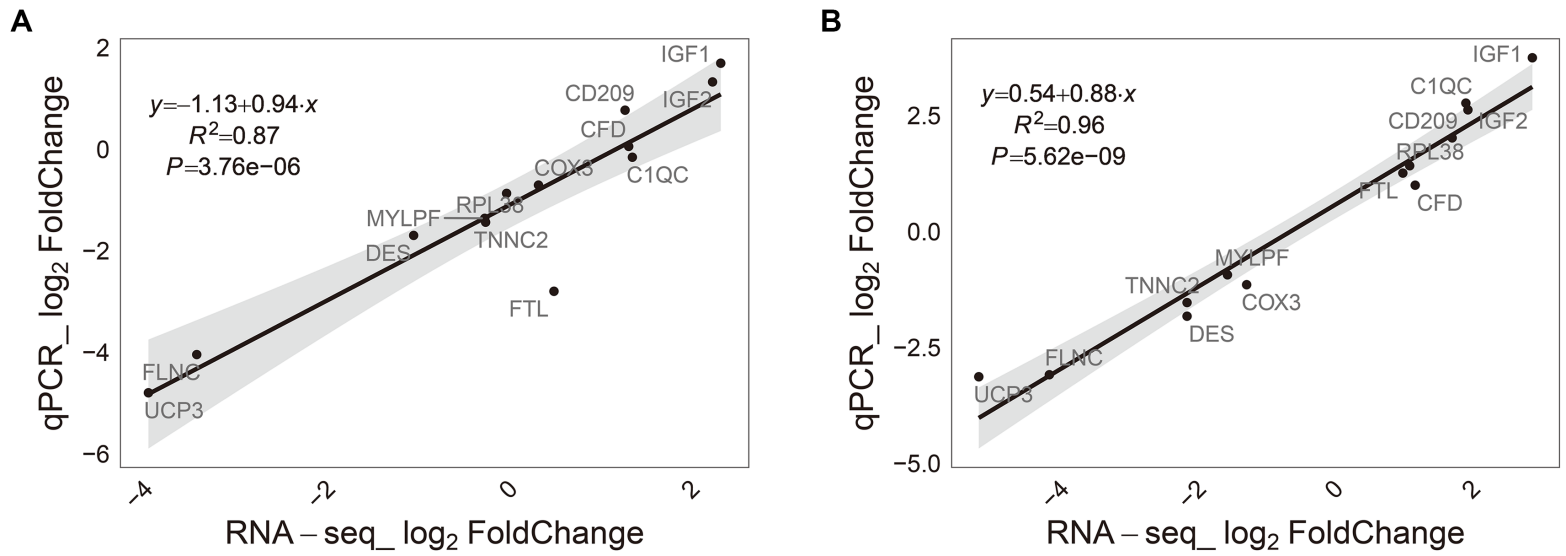
Consistency of RNA-Seq results and qRT-PCR. **(A)** Muscles (*n* = 4). **(B)** M-EVs (*n* = 4). M-EVs, Muscle-derived EVs.

To validate the RNA-seq findings, the expression of 13 differently expressed genes (DEGs, with a |fold change| ≥ 2 and adjusted *p*-value ≤0.05 in M-EVs vs. muscles) in M-EVs and muscles were determined by quantitative real-time polymerase chain reaction (qRT-PCR). Correlation analysis demonstrated strong and significant correlations between the gene expression levels obtained from qRT-PCR and those from RNA sequencing in M-EVs (*r* = 0.97, *p*-value <0.001) and muscles (*r* = 0.93, *p*-value < 0.001). The results confirm the accuracy and reliability of the differential expression profiles observed through RNA-seq (Figures 7A,B).

## 4 Discussion

Initially, EVs were considered as “platelet dust” in plasma by Peter Wolf detected (32). However, their significance shifted dramatically after the discovery of RNAs in EVs (EV-RNAs) in 2007 (33), which sparked growing interest among researchers. EVs are now acknowledged for their pivotal role in intercellular communication, facilitating the transfer of biological active molecules between cells (34). Researchers have initiated investigations into the variation in EV-RNA composition in body fluids across different species, and certain EV-RNAs have been found to be conserved among them. A comparative study of miRNAs in milk EVs from human, cow, porcine, and panda unveiled the preservation of several abundant miRNAs, such as let-7a, let-7b, let-7f, and miR-148a, with sequence homology between species. These miRNAs have been implicated in immune functions, cell growth, and signal transduction (35). In addition, differences in resistance characteristics between breeds may be reflected in EV-RNAs. For instance, exosomal miRNAs in milk and colostrum of Doğu Anadolu Kirmizisi (DAK) cows were more associated with immunological pathways compared to Holstein cows, suggesting that DAK cows are more resistant to harsh environmental conditions (36). It is important to note that most of these studies have primarily focused on exploring differences in EV-RNAs between species or breeds. Only Mecocci et al. have discussed the potential role of EV in generating phenotypic traits that distinguish species (37). Therefore, delving into the realm of EV-RNAs holds great promise, as it may uncover unclear mechanisms mediated by EV-RNAs that contribute to the development of specific phenotype in domestication.

Furthermore, it is essential to recognize that EVs isolated from body fluids alone may not accurately reflect the true physiological state of the body, necessitating a focus on tissue-derived EVs (38). Unfortunately, research on tissue-derived EVs in pigs remains limited, with Xiong et al. being the only ones to explore non-coding RNA in porcine anterior pituitary derived EVs (39). In this study, we surveyed the mRNA expression profiles of muscles and its derived EVs in Large White pigs and wild boars, and investigated the heterogeneity of enriched genes in M-EVs in terms of number and biological functions.

Analysis of the mRNA expression profiles between M-EVs and muscles revealed a low correlation of mRNA expression between M-EVs and muscles (r-mean 0.78), suggesting that M-EVs do not exactly replicate the expression profiles of muscles, which is consistent with previous reports (40). However, we also found a stronger tendency for low-expressed genes to be enriched in M-EVs compared to high-expressed genes in muscles, suggesting the existence of a mechanism for active and selective mRNA packing by M-EVs. Moreover, it has been observed that M-EVs exhibit a lower number of reads aligned to the CDS region and a higher number of reads aligned to the UTR region in comparison to muscles. This observation is in line with previous reports (40, 41), which suggest that EVs not only deliver intact mRNAs for translation in target cells (4), but also carry UTR sequences to regulate the stability, localization, and translation activity of endogenous mRNAs within those cells (5).

Cyclin D2 (CCND2) is an important cell cycle regulator that affects cell proliferation and development by mediating the G1 to S phase transition (42–44). In mouse and porcine models of myocardial infarction, up-regulation of *CCND2* expression promotes cardiomyocyte proliferation, reduces myocardial infarct area, and improves cardiac performance (45). In contrast, in mouse myoblasts, down-regulation of *CCND2* enhances their myogenic differentiation by inhibiting the phosphorylation of retinoblastoma protein (pRb), which produces a greater number of myofibrils than down-regulation of the myostatin gene-induced myogenic differentiation (46). We found that *CCND2* was enriched in M-EVs of wild boars and Large White pig, suggesting that it may have the potential to maintain cell type homeostasis in muscle tissue or to regulate distal cell proliferation.

Immune responses are primarily classified into innate and adaptive immunity (47). In innate immunity, complement and coagulation are blood-related protein hydrolysis cascades, wherein the coagulation cascade can limit bleeding and heal wounds, while the complement system serves to eliminate pathogens and damaged cells (48, 49). In adaptive immunity, antigen processing and presentation is the cornerstone of this immune response (50). Extracellular vesicles have been shown to be important players in the progression of inflammation, antigen presentation, B and T cell development and activation in both innate and acquired immune responses (51). We found that genes enriched in M-EVs from wild boars and Large White pigs were co-enriched in KEGG pathways associated with innate immune responses, such as Complement and coagulation cascades and Fc gamma R-mediated phagocytosis. This suggests that muscle tissue may be involved in the organismal immune response through the release of EVs. The Notch signaling pathway is an evolutionarily conserved pathway that induces the proliferation and differentiation in stem cells into distinct terminal cells in body development and injury repair (52). Similarly, the TGF- beta signaling pathway is widely recognized for its crucial role in regulating embryonic development, tissue homeostasis, and damage repair. It has been observed to affect a wide range of cells, thereby contributing to the maintenance of overall tissue homeostasis (53). Notch and TGF- beta signaling proteins have been found to be sorted into exosomes, which have been shown to promote cell proliferation, differentiation and activity (54–56). In contrast to the genes enriched in M-EVs of Large White pig that were specifically enriched to the immune-associated KEGG pathway, we found that the genes enriched in M-EVs of wild boars were specifically enriched to pathways with a wide range of functions (regulation of immunity, cell fate, hormone release, and metabolism, etc.), including the Notch and TGF- beta signaling pathways. Thus, it is plausible to speculate that M-EVs may potentially regulating skeletal muscle growth or influencing biological processes of other tissues through carrying mRNA. Also, these results indicate that the mRNAs enriched in the M-EVs of wild boars are more diverse in type and function than those in the M-EVs of large white pigs.

Although the results of our study are intriguing, we acknowledge their limitations due to the modest number of animals involved. Additionally, as EV extraction technology is continuously developing, this potential limitation should be considered. Further studies on EV-mRNAs secreted by muscle are needed, including investigations into the sequence integrity, protein coding abilities and physiological functions.

## 5 Conclusion

In this study, we investigated the different mRNA expression profiles between M-EVs and muscles using next-generation sequencing, and further explored the heterogeneity of enriched genes in M-EVs between Large White pigs and wild boars regarding number and biological functions. The results indicate that intracellular mRNAs are enriched in M-EVs, especially mRNAs that are micro-expressed in the muscles. Meanwhile, compared to muscles, only a portion of mRNAs in M-EVs are full-length and contain the CDS region. On the one hand, these enriched mRNAs in M-EVs show commonalities between the two breeds, e.g., the genes enriched in M-EVs from the two breeds are co-enriched for the innate immunomodulation-related KEGG pathway. On the other hand, heterogeneity between the two breeds was also fed back, e.g., compared to the Large White pigs, the genes enriched in M-EVs from wild boars were specifically enriched to KEGG pathways affecting cell fate such as the Notch signaling pathway and the TGF-beta signaling pathway.

## Data Availability

The original datasets generated by sequencing presented in the study are deposited in the Genome Sequence Archive (GSA) repository (https://ngdc.cncb.ac.cn/gsa/), accession number CRA019086.
