# Supplementary material for: mRNA expression profiles in muscle-derived extracellular vesicles of Large White and wild boar piglets reveal their potential roles in immunity and muscle phenotype
Source: Front Vet Sci. 2024 Oct 3;11:1452704. doi: 10.3389/fvets.2024.1452704 (PMC11484452; doi:10.3389/fvets.2024.1452704)
Supplement: Supplementary file 1 [file Data_Sheet_1.zip › Supplementary Material_V2/Additional file 1.pptx]

## Slide 1
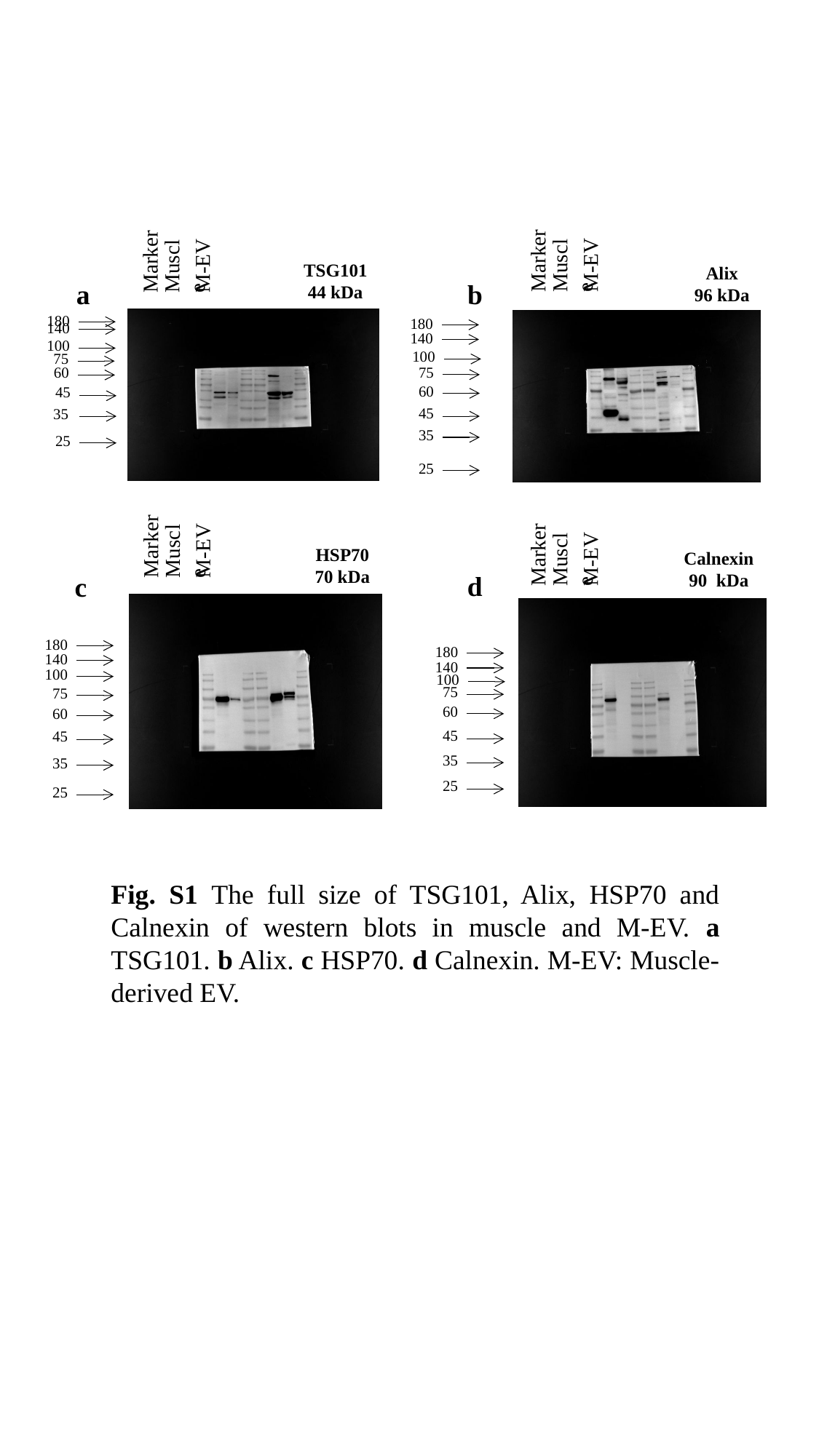

Marker
Marker
M-EV
M-EV
Muscle
Muscle
TSG101
44 kDa
Alix
96 kDa
b
a
180
180
140
140
100
100
75
75
60
60
45
45
35
35
25
25
Marker
M-EV
Muscle
Marker
M-EV
Muscle
HSP70
70 kDa
Calnexin
90 kDa
d
c
180
180
140
140
100
100
75
75
60
60
45
45
35
35
25
25
Fig. S1 The full size of TSG101, Alix, HSP70 and Calnexin of western blots in muscle and M-EV. a TSG101. b Alix. c HSP70. d Calnexin. M-EV: Muscle-derived EV.
